# Challenges for the smoking ban in Israeli pubs and bars: analysis guided by the behavioral ecological model

**DOI:** 10.1186/2045-4015-1-28

**Published:** 2012-07-24

**Authors:** Orna Baron-Epel, Carmit Satran, Vicki Cohen, Anat Drach-Zehavi, Melbourne F Hovell

**Affiliations:** 1 School of Public Health, Faculty of Social Welfare and Health Studies, University of Haifa, Mount Carmel, Haifa, Israel; 2 Department of Nursing, Faculty of Social Welfare and Health Studies, University of Haifa, Mount Carmel, Haifa, Israel; 3 Center for Behavioral Epidemiology and Community Health, Graduate School of Public Health, San Diego State University, San Diego, CA, USA

**Keywords:** Smoking ban, Bars, Pubs, Behavioral ecological model, Israel

## Abstract

**Background:**

The latest amendment to the ban on smoking in public places in Israel was implemented in 2007, adding pubs and bars (P&B) to the list of public places in which smoking is prohibited. However, smoking in most P&B continued. The aim of the study was to identify the theoretically plausible reasons for the partial success of a public ban on smoking in P&B settings. Explanations provided by P&B owners were interpreted as probable causal factors based on the Behavioral Ecological Model (BEM).

**Methods:**

Qualitative interviews were performed with 36 P&B owners in Tel-Aviv and 18 Israeli towns and cities of various population size.

**Results:**

P&B owners reported a variety of situational factors (*i.e.*, contingencies) and reinforcers as likely explanations of the partial failure of the legislated ban on smoking in public places, particularly P&B. The major reinforcers for non-adherence with the law were no or low frequency of inspections and low penalties from authorities. P&B owners also feared loss of customers and revenue if bans were enforced in their own establishment but not in competing establishments. Finally, owners reported social norms prevailing among some Israeli patrons supporting smoking in P&B settings, in part to express opposition to the new law.

**Conclusions:**

Qualitative assessment can uncover probable social situations that operate to prevent greater adherence to smoking bans. The results warrant confirmation by quantitative analyses. Policies with mandated inspections and penalty requirements that are implemented in all bars without prejudice could lead to greater adherence to smoking bans. Positive reinforcing consequences that encourage adherence (such as publicity and support from non-smokers) would be more likely to generate both greater adherence to the policy and good will toward the government. Principles of behavior outlined in the BEM offer guidance for designing quantitative confirmation analyses of future bans.

## Background

Many countries have enacted laws banning smoking in public places as part of their strategies to decrease the ill effects of smoking. This strategy has reduced smoking and levels of exposure to second-hand smoke (SHS) in many countries [1-3]. In recent years some countries and cities have banned smoking in pubs and bars (P&B), further reducing exposure to SHS [2,4-8].

In 1983, a law banning smoking in public places in Israel was enacted and amendments to the basic law were added over the years. The latest amendment came into effect on November 7, 2007, adding P&B to the list of public places in which smoking is prohibited, except in designated areas, when they exist. This amendment placed the responsibility of enforcing the smoking bans on the proprietors and the smoking patrons. Proprietors are fined 5000 NIS, about $1250, and the smoking patrons are fined about $300. Rosen and associates [9] measured respirable small particles (RSP) in P&B and cafes in Israel before the enactment of the new amendment. They found that the average level of RSP was about 10 times higher than in countries with enforcement of smoking bans and similar to countries without enforced smoking bans. After the enactment of the law, these authors reported that while the levels decreased somewhat, they were still very high according to EPA standards, especially in Tel-Aviv [10]. As levels of smoking in P&B seem to be high, even after enactment of the ban on smoking in these settings, it is important to understand the conditions that determine the effectiveness or non-effectiveness of a ban.

The Behavior Ecological Model (BEM) was developed to extend the theory based on conditioning of behavior to explanations of group and population practices [11,12]. As an individual’s health is usually a function of the behavior of many people on multiple levels (individual, community, and national), a broader systems approach to understanding the individual and population behavior is critical to identifying ways to improve health associated with the behavior of large groups and whole populations. Complex systems of behavior of subgroups within the overall population operate to influence complex behavioral and health outcomes. Systems influencing behavior take place at the individual level of analysis, family and peer network levels, and at larger community and even international levels. The BEM relies on concepts and principles of behavior in which rewarding or punishing consequences that immediately and reliably follow a given behavior influences the adoption of the behavior [13,14]. Causes of behaviors are to be found in the environment external to the phenomenon to be explained. Such contingencies (defined as a situation determining a behavior) either increase or decrease future behavior, depending on the power of the reward and other context factors [11,12].

These contingencies of reinforcements define complex ecological social systems that may predict and might inform new policies and other interventions to establish population-wide health practices, including no smoking policies in public places [11,12]. This model seems appropriate for the complex issues of smoking in P&B as it focuses on both the individual's behavior and on populations and individuals that have an influence on individuals, such as bar owners and local authorities. Other models such as the Health Belief Model or the Theory of Reasoned Action and Planned Behavior do not encompass all levels of society that are needed to cope with complex behavioral issues such as the ban on smoking in P&B. These models relate to the individual and interpersonal levels of society. Hovell and Hughes [11] suggest that successful use of the BEM to formulate effective policies, together with effective enforcement to protect nonsmokers from SHS exposure, could lead to the eradication of most tobacco smoking.

Hovell and Hughes [11] have interpreted the BEM to suggest how social norms may be changed by restricting smoking in public settings and how this might also markedly change social norms with respect to tolerating tobacco smoke. One of the most important themes in their paper suggests that cumulative regulations that increasingly limit micro-environments where smoking is allowed will lead to less smoking and an increase in the population’s intolerance of smoking in all environments.

The anecdotal reports of low levels of enforcement of the law banning smoking in P&B in Israel serves as an interesting social context to study possible social contingencies that may explain relative adherence or non-adherence to bans in P&B, and may help develop policies that will ensure greater adherence to such bans.

## Methods

### Study design

Qualitative semi-structured interviews were conducted with 36 P&B owners during January to August 2010. This study is part of a larger study assessing factors influencing the implementation of the ban on smoking in P&B in Israel, such as levels of airborne nicotine in the bars, the bar's environment, attitudes of the clients in the bars, and attitudes of local authority officials.

At this point in time the factors that influence P&B owners’ adoption or non-adoption of the law were unknown; thus it was necessary to identify these issues in a qualitative study before being able to measure them in a quantitative study.

Thirteen P&B owners were from P&B in Tel-Aviv (a large metropolitan city in Israel); the other 23 P&B owners were from 18 towns around Israel. In Tel-Aviv the competition between bars is substantial; however in the smaller towns there is very little competition. In Tel-Aviv, the P&B were chosen randomly in order to gain representation of various types of bars. Bars in the other towns were chosen first by identifying towns with 20,000 to 220,000 residents having a majority of Jews, and with P&B in their jurisdiction. Arab towns were not included as P&B are not a common venue for entertainment among Muslims as alcohol is not allowed. The selected group included 32 towns; however, only 29 were included in the large study as three towns were near the Gaza strip, which at that time were unsafe to visit. Most towns had only one or two bars and the choice of towns and bars for the final interviews were based on convenience. However, the realized sample includes more than half of all the towns eligible for the study. Seven interviews were not completed due to failure to find the owner, refusal, or postponements of appointments. A total of 36 interviews were completed.

Each interview took 20 minutes to an hour, depending on the willingness of the bar owners to share their opinions and experiences with the interviewers. The potential interviewees were approached by phone or in the bar; the aims of the study were explained and a date was made for the interview. The interviews were transcribed verbatim from recordings or transcribed during the interview. Some interviewees refused to be recorded; however we do not think this biased the data as similar themes and categories were identified in both types of interviews. A topic guide led the interviewee through the issues for discussion. The guide addressed such issues as smoking status of the bar owner, age, type of P&B, attitudes towards the ban on smoking in P&B, what the policy of the pub was regarding smoking, experience with enforcement of the law, experiences with patrons related to the law, and how they cope with the patrons and the authorities. The sample size was reached after no new themes or categories were identified.

The study received approval from Haifa University’s ethics committee before beginning the interviews.

### Analysis

Data were analyzed according to the guidelines of Unrau [15]: the text was divided into meaningful units, its categories were identified, and finally the data were explained and interpreted. Three of the authors read the transcripts and each team member coded and organized the data to identify key categories. The lists of categories were compared, and any inconsistencies were resolved by a review of the data and then reaching agreement through discussion. The researchers then reread all the transcripts and found the themes running through the interviews [16]. The same process was used to obtain a consistent list of themes. The BEM was used as the conceptual framework for which categories and themes were interpreted to arrive at plausible hypotheses explaining the continued smoking in P&B in the context of a ban on such behavior. Categories were divided by the hierarchy of contingencies that reinforced the behavior of smoking in P&B; if the category reinforced smoking in P&B generally in the whole society it was categorized as such, and if it reinforced smoking in a specific P&B it was classified as belonging to the local level, and so forth.

## Results

Table 1 presents the characteristics of the 36 P&B owners interviewed. The mean age was 32 and 89% of the owners were men; over half of the owners smoked. The P&B owners were from small to large towns; 44% of the P&B owners did not hesitate to report that they did not enforce the law. Most of the remaining implied that they sometimes adhered to the law by enforcing it “as best they could”.

**Table 1:** Bar owners’ characteristics, number, and percent

		**Number (percent) of pubs/bars or owners 36 (100%)**
**Mean age**	32 years Range 23–47	
**Gender**	Male	32 (89%)
	Female	4 (11%)
**Smoking status**	Present smokers	20 (56%)
	Past smokers	3 (8%)
	Never smoked	13 (36%)
**Self report of ban enforcement in the bar/pub**	Enforce the ban	14 (39%)
	Partially enforce the ban	6 (17%)
	Do not enforce the ban	16 (44%)
**Size of town or city where the bar/pub is located**	Tel-Aviv (400,000 residents)	13 (36%)
	Towns with 220–100,000 residents	7 (19%)
	Towns with 100,000–50,000 residents	4 (11%)
	Towns with 49,000–25,000 residents	12 (33%)

Table 2 depicts the themes and categories by hierarchy for which we analyzed the interviews. The two main themes that emerged were: 1. Financial and social reinforcements and punishments for enforcing or not enforcing the ban; and 2. Situations enhancing or prohibiting adopting the ban (contingencies).

**Table 2:** Themes and categories identified from interviews with the bar owners by hierarchy of contingencies

**Themes**	**Financial and social reinforcements for punishments for adopting the ban**	**Situations enhancing or prohibiting adopting the ban**
**Level of Hierarchy**	**Category**	**Category**
1. Society level	1. The social perceptions of “having fun” in the bar.	1. Banning smoking as a result of social criticism.
		2. Reactions to social movements trying to enforce the law.
		3. The disobedient Israeli customer.
2. Community level	1. Frequency of receiving fines.	1. Inconsistencies in inspectors’ behavior.
	2. The courts as a non-supportive organization environment.	
3. Local level	1. The bar’s losses and gains in enforcing the ban.	1. Inconsistencies in adherence to the ban.
4. Individual level		2. Problems in the built environment preventing adherence with the ban.
		1. Patrons’ reactions to smoking in P&B.
		2. The bar owner’s role as the enforcer of the ban.

### Financial and social reinforcements and punishments for adopting the ban

#### Society level

##### The social perceptions of “having fun” in the P&B

The P&B owners think their clients expect a certain atmosphere in the P&B and do not want to be told what to do when they are out having fun. Therefore, they think the ban will change the atmosphere clients expect:

“This is some kind of an escape … it is not a spa or a gym, people come here to drink, it is not a healthy place, it’s an escape that symbolizes the anti-climax of health”

“People go out to a bar for a drink and smoke a cigarette. They want to get away from the daily routine for an hour or two … It’s the last place that you can still have some fun these days …”

It seems that P&B serve to relax social and legal rules about drinking and smoking in order for patrons to have “fun”. This implies that owners may be criticized or lose business if they penalize patrons for smoking.

### Community level

Local authorities are the law enforcers of the ban on smoking in public places; their inspectors are expected to enforce the law by giving fines.

#### Frequency of receiving fines

The P&B owners reported different levels of law enforcement depending on the local authorities. Many P&B owners reported no inspections of the B&P.

“You can’t see enforcement in Israel at all.”

“The inspectors go to sleep after 12.30, they don’t work on Saturday”

On the other hand there were P&B that reported having being fined:

“In (name of town) the law is enforced; there are inspectors and they enforce it mainly in my bar! I am located near the city hall … so they start and finish their rounds here.”

One bar owner said:

“The inspectors come every day, they drive me crazy.”

P&B owners had the option to call the city hall’s call center and complain if clients smoke in the bar; this served as a way to get round a fine if the inspector came. However one bar owner said:

“I called the call center and asked them to send an inspector when the law just came out, but as time went on they ignored my calls and stopped coming, so we stopped calling.”

The low level of inspection and financial penalties made it unlikely that the “no smoking” ban would be enforced. Theoretically, inconsistent punitive contingencies generate gambling and may have inadvertently increased the probability of non-adherence to the law. Moreover, the possibility of a wide-spread impression that the law was enforced in a prejudicial or an unfair manner might lead to a thwarting of the law or even overt counter-aggression.

#### The courts as a non-supportive organizational environment

The P&B owners described situations where they were sued; however, the courts did not penalize them with large fines. On the contrary, they were fined for low sums of money or did not appear in court at all.

"Twice I was taken to court, once it somehow ended and once I lost the case but the judge said they can’t be greedy and I was fined only 150 shekels (around $50), even though I was sued for thousands of shekels."

"They close the case for 'no interest to the public'; they do not take the case to trial so it will not turn into a precedent."

These reports suggest that there is no overall social agreement as to the enforcement of the law; the courts do not back the individuals or organizations that try and enforce the law, while the local authorities refrain from fully inspecting the P&B.

### Local level

#### The bar's losses and gains in enforcing the ban

##### Financial losses for enforcing the law

Some of the P&B owners reported real or perceived financial losses due to loss of clients when trying to enforce the law.

"… as a bar that was founded before the law we had a 30% decrease in revenues …"

"People come in and say: What? You can’t smoke here? and they leave."

"If the barman tells someone not to smoke, his tips will decrease."

Some P&B owners reported financial losses due to the fines they had to pay:

"We paid 15,000 shekel (about $5000) already … that’s a lot of money."

#### Gains in enforcing the law

A few P&B owners who enforce the law at least partially, said that clients who do not want to suffer from SHS come to their bar, and they are rewarded when the clients praise them for a clean air P&B. In addition, having a "clean bar" brings in a specific set of patrons:

"I hear mainly positive remarks, especially from the girls, 'It is so good not to stink when we go home, no need to wash your hair' …"

"… we market the bar as a non-smokers bar so we get non-smoking clients."

Both losses and gains serve as reinforcement of the behavior adopted by the P&B. P&B not enforcing the law perceive not losing clients and revenues as reinforcement for not enforcing the law, while those enforcing the law perceive the satisfaction of the non-smokers as reinforcement for adhering to the law.

### Situations enhancing or prohibiting adopting the ban (contingencies)

#### Society level

##### Banning smoking as a result of social criticism

P&B that served food during the day and the early hours of the evening reported implementing the law when their main business was food. Later in the evening they did not enforce the law. This implies that they adapted their establishment to a specific social contingency:

"There is a separation between noon and night; during the day there is no smoking also outside. I had a problem with families during the day, that’s why it turned into non-smoking (establishment). We go by the norm, and tailor for our target population."

These comments suggest that social criticism and risk to their business was driving their enforcement of the “no smoking” ban for food services in the daytime but not for drinkers in the evening.

#### Reactions to social movements trying to enforce the law

Some P&B owners in Tel-Aviv mentioned an organization called "Clean Air", an NGO founded by law students who advocate for smoke-free public places, mainly P&B. This NGO is active only in Tel-Aviv. Their members usually go to P&B, take pictures of smoking clients, and sue the P&B. The P&B owners said that most of their appearances in court due to smoking were instigated by this organization. However, the judges were not pro "Clean Air". This organization was perceived by the P&B owners as "informers" and "anti social":

"It is obvious that their intentions are not pure, the person comes with a camera and harasses people …"

"The judges don't like them (Clean Air)."

As they perceive these activities are not backed by the various institutions, such as the courts, the P&B owners did not report trying to enforce the law due to the NGO's activity. P&B owners in the other towns did not mention such organizations.

#### The disobedient Israeli customer

During the interviews comparisons to the ban on smoking in P&B in other countries came up and the P&B owners tried to explain why it was so hard to implement it in Israel. In their opinion the Israeli client is "problematic": they thought it was impossible to make Israelis change their behavior and obey the law; the law will not prevent the patrons from doing what they want to do. This is seen as a social norm among Israelis.

"…people do not listen, they just light the cigarette later (after being told not to smoke); even next to the inspector they go on smoking."

This perception of the disobedient Israeli may serve as an excuse for not enforcing the law, but it may also be possible that Israelis perceive themselves as oppositional to laws and do not appreciate criticism of their smoking practices.

### Community level

#### Inconsistencies in inspectors’ behavior

In Tel–Aviv, where there are over 150 P&B and there is fierce competition, the P&B owners complained of unequal enforcement of the law by the authorities. In the opinion of P&B owners, the inspectors preferred to inspect certain areas and certain types of establishments but not others. These P&B owners felt they faced unfair competition with other P&B where the law was not enforced, and where the competition was not fined or sued. The bar owners expressed high levels of frustration:

"The law is enforced where it is convenient for them …"

"They are terrible, they are bad at anything they do, I don't even know where to start. The inspection division (in city hall) is run like the mafia, with revenge on a personal level, they bother the weak, and fear the strong, … can't you (the authorities) solve the problem? When you want to, you can!"

"… once a month they (the inspectors) come and we get fined … I have not heard of anyone else in the area getting fined …"

The unequal enforcement may lead to non-adherence with the smoking bans.

### Local level

#### Inconsistencies in adherence to the ban

Bar owners complained of nearby bars where clients can smoke:

"My neighbor (a bar) does not enforce the law, so I have no financial interest in enforcing the law …"

These reports emphasize the importance of a total ban in all bars; this is crucial when trying to achieve compliance with the law, as previously mentioned.

#### Problems in the built environment preventing adherence with the ban

Some of the P&B owners complained that technically they cannot have smoking areas; thereby making it harder for them to compete with bars that have smoking areas:

"Our place is a studio. We cannot separate it for smokers and non-smokers so we let the customers smoke in the whole area … we can't do anything about it …"

If a P&B is non-smoking, the clients will smoke outside the bar; however, in a residential area the bar owners are at risk of getting their permit taken away as they are disturbing the residents by having people make noise outside on the sidewalk:

"If I send him (the client) to smoke outside I am taking a risk of getting fined for disturbing the neighbors, and that puts my license at risk; smoking in the bar is just a fine, it does not put the whole business at risk."

They also thought that when smokers had to go outside to smoke the place looked empty and the atmosphere was negatively affected.

### Individual level

#### Patron's reactions to smoking in bars

The P&B owners reported that only infrequently did clients complain about smoking or told other people in the bar to stop smoking.

"Only a few times I heard clients comment about the smoking; usually they do not complain."

P&B owners had stories of policemen and inspectors who smoked in the bar; this presented a negative role model for the public.

"… a few days ago a policeman came in and smoked … when there is no enforcement there is no enforcement."

#### The bar owner's role as the enforcer of the ban

The law created a situation in which instead of being a host, the bar owner perceives himself as a "policeman". This puts him in very difficult situations with clients.

"… the law turns me into a policeman … I can't be a policeman …"

"The bar owners' job is to make a bar fun, not to be a policeman."

"I won't be the bad guy …"

It seems that the P&B owners have no intention of taking on the role of law enforcement.

## Discussion

### International bans on smoking in P&B

Involuntary smoking is known as the third highest risk factor for mortality throughout the world, causing cancer, heart disease, and severe and chronic respiratory diseases [17-19]. A smoke-free policy banning smoking in public places is the most efficient strategy to reduce involuntary smoking and to eliminate the ill effects of SHS [1,20,21]. In most developed countries there are policies that ban smoking in certain public places; in some countries the ban is comprehensive and in others it is partial [18]. It seems that a total ban is more effective than a partial ban [1,22]. Ireland was the first country to ban smoking in all public places including P&B; this was the last public setting to prohibit smoking as alcohol and smoking were perceived as associated behaviors [4,23,24]. Other countries followed and studies show a large decrease in SHS in the P&B after enactment of the bans [6,22,24,25].

Studies also compared P&B in countries with total bans and countries without bans on smoking in P&B. The levels of airborne nicotine in the P&B in countries with the bans were 93% lower than in countries that do not ban smoking in P&B [22]. Most countries implementing these bans have been successful; however, not all. For example, in Poland and the Slovak Republic airborne nicotine was still not very low [24]. In Australia, before banning smoking in P&B Carter & Chapman [26] reported that laypeople considered smoking in bars "natural" and thought venues would fight bans; this was expressed more frequently by smokers. However, Cooper *et al.*[27] found that smokers readily comply and supported smoke-free bars after enactment of the law. In Greece, young people were cynical about government and thought legislation would be ineffective [28]. In Israel, it seems that even though a law banning smoking in P&B was enacted, not all establishments adhere to it [9,10]. In the annual report to the Knesset (Israel parliament) towns were asked to report on their law enforcement. Forty-one towns reported on the number of fines given out because of smoking in public places; in Tel-Aviv 1343 fines were given out in 2010, and in the other 40 towns another 2,244 fines were issued [29]. Comparatively, this is a relatively small number of fines, implying a low level of law enforcement in Israel.

### Analysis using the BEM

Analysis of the P&B owners' narratives may help us understand how social norms regarding smoking in P&B may be changed by altering contingencies of reinforcement using the BEM.

Situations in which reinforcements and punishments promote enforcing the law in the P&B by the owners were identified for each level of hierarchy in society. This includes the society, community, local, and individual levels, which form a synergy determining the actions of the P&B owners. In this way it may be possible to develop a society with norms that are not tolerant of smoking in public places and where society will provide social reprimands that will prevent the smokers from smoking in public places. Hofstetter and colleagues [30] suggested it is plausible that the presence of policies banning smoking will function as motivating reprimands and sanctions from non-smokers towards the smokers, and will help develop a non-smoking society. However, to start the process more substantively in Israel, it may be necessary to strengthen laws and their universal enforcement.

According to the P&B owners' narratives, the various levels of hierarchies in society provide contingencies and reinforcements for not enforcing the law and do not provide substantial punishments.

From the narratives two main categories of reinforcements and punishments can be identified and each has various contingencies defining the behaviors. The main reinforcement or punishment is financial: receiving fines from the local authorities' inspectors, losing in courts when being sued, and loss of customers who want to smoke. The second is the social reinforcement and punishment.

### Financial reinforcement or punishment

Infrequent and inconsistent inspections result in a lack of effective reinforcements and punishments, which can be the result of different reasons, such as not enough funding for inspectors, or low priority within the local authority. In addition, the inspectors may use their power unfairly and/or for personal gain, even for vengeance, as suggested by one bar owner. Infrequent and inconsistent inspection generates serious dissatisfaction with the no smoking law and criticism of the authorities, discouraging any attempt to fully enforce the law. Intermittent reinforcement can sustain behavior indefinitely. However, typically unreliable punishment does not reduce rates of unlawful behavior. Sometimes it strengthens behavior due to intermittent reinforcement. The net picture provided by our interviews suggests very limited penalties for smoking in P&B, making such procedures unlikely to suppress smoking. The reported Infrequent and inconsistent inspections of the P&B may imply that if they would perceive the inspection as consistent, enforcement of the law would be possible.

Laws that depend on penalties should also have relatively large fines that increase with repeated offenses. However, because inspectors are uncomfortable assigning high cost penalties, they will require training and supervision to ensure the universal application of penalties when earned. It is important to increase the knowledge of inspectors regarding the hazards of SHS and to convince them that they are saving lives when they execute a fine.

Support for these findings can be obtained from the annual report to the Knesset regarding the low number of fines given [29], which implies a low level of law enforcement, correlating with the P&B owners’ reports.

Environments that were non-supportive of the financial penalties were identified. For example, the judicial courts did not support suing the P&B; therefore, laws should not allow judges to depreciate the use of the law in general or the level of penalty. Another unsuccessful contingency on the society level was the "Clean Air" organization. From the reaction to the social movement "Clean Air", it seems that the P&B owners do not understand the reasons why this social movement exists; their narrative implies that they think the aim of "Clean Air" is somehow to profit financially. It seems also that this organization misses the point they are trying to make, as their "harassment" of the P&B does not induce the implementation of the non-smoking policy. This fits the cultural contingency model that Hovell *et al.*[12] have proposed as powerful at the society level.

The P&B owners also think that smoking increases alcohol consumption; therefore a smoking ban will cause patrons to order less alcohol. These issues have not usually been found to be true in formal studies [31-33]. In addition, the fact that the bar owners think that there will always be a nearby bar where clients can smoke prevents them from enforcing the law, as they presume that clients will prefer a bar that does not enforce the law. Of course this is mainly true for P&B that have competition nearby. It seems that the P&B owners' perceptions and emotional reactions to risks of loss of income are more powerful than evidence to the contrary. Their perception of the issue seems to be preventing them from enacting the ban. This perceived risk is also why it is important that new laws are well advertised and social marketing techniques adapted, and that all P&B that break the law consistently experience the same penalty.

The P&B owners' perceptions that most patrons are smokers inhibit them from analyzing the situation more accurately. In Israel, as in most countries, there are more nonsmokers than smokers (even among those who frequent bars) [34,35]. This suggests that the P&B who cater to non-smokers could earn more than from smokers. However, many nonsmokers appear to put up with the smoke because they otherwise can’t go anywhere to enjoy an evening out and do not shun P&B not enforcing the ban.

### Social reinforcements and punishments

Social reinforcements and punishments and situations that provide these reinforcements can be identified at all levels of analysis. For example, on the society level, subsets of customers do not want to eat or drink in the presence of smoke. This has reinforced the P&B owners’ adherence with the law to restrict smoking during early hours of operation. In the evening when patrons come only to drink the P&B owner relaxes the ban. This discrimination on the part of some owners is most informative and shows that owners do respond to the wishes of their customers. It suggests that the complaints and/or loss of business by subsets of the public have led to changes in P&B owners' enforcement of the law in their P&B. It is possible for financial and social contingencies to alter the owners’ enforcement of policies. However, on the other hand, the P&B owners said that P&B are for patrons to have a good time, enjoy the music, drinks, and company; this includes both alcohol and smoking. This implies a complex and subtle reinforcer and contingency. With a ban, the P&B owner must criticize some customers when asking them to stop smoking and this probably makes the customers uncomfortable. Thus, the implied fines in existence may not be enough to make it worth the risk for bar owners to enforce the law, given the social penalties that might take place, such as detracting from the desired atmosphere in the P&B. On the individual level, it seems that the non-smokers present very little reinforcement for adopting a non-smoking policy in the P&B, as in the evening they are less inclined to express their dissatisfaction when the P&B allows smoking.

### Specific contingencies

Contingencies that support or prevent the enforcement of the ban were identified for the different hierarchies of society. One such social contingency mentioned by P&B owners was the disobedient Israeli; the owners do not want to confront the smokers as it may bring on negative reactions by the patrons. The notion that the state laws can be bypassed and the system can be beaten has been discussed and studied by various scholars in Israel. Sprinzak [36] suggests that the disobedience in the Israeli society stems from the Jews having spent centuries in the Diaspora, where they viewed the laws as alien. "Beating" or "bashing" the system is regarded as normative in certain groups. The issue was discussed and empirically studied [37]. For example, Rattner *et al.*[38] studied three groups of Israelis: Ultra-orthodox Jews, settlers in the occupied territories, and Israeli Arabs. They suggested a model predicting willingness to take the law into one's hands, where commitment to the law, perception of procedural justice, and alienation were the independent variables.

However, this does not mean laws cannot be enforced; they do need strong and consistent enforcement together with large social marketing campaigns as described for prevention of road accidents [39] and smoking bans in other countries [4,5]. Implementation of the ban on smoking should not depend on the P&B owners, and law enforcement cannot be left to them with no reinforcements for their actions.

Another such contingency that the bar owners mentioned was the fact that the law does not take into account specific problems they have that prevent them from having smoking areas such as small and old bars; these may be serious problems that should be addressed in refined policies.

## Conclusions

The lack of consistent use of various punishments and reinforcers inhibit the implementation of the ban on smoking in P&B in Israel. From the owners' narratives it seems that consistent and equal inspection of the P&B, in addition to the backing of the judicial courts, together with a campaign targeting the non-smokers with a message that they are entitled to a smoke-free environment in all public places including P&B could change social norms. Social norms could be supportive of not smoking in P&B and other public places. It seems that the P&B owners have a constant conflict of interest; on one hand they would like to be law abiding citizens, but on the other hand, abiding the law brings with it too many punishments that do not increase the likelihood of enforcing the law. At this point in time, the reinforcers for not implementing the ban are more influential than the punishments at all levels of social hierarchy.

When the law was enacted, the P&B owners were expected to comply with the law. Some prior educational information regarding the law was communicated *via* the radio and direct mailing to P&B owners; however little information regarding how to tackle the problems that would arise was given. No budget was allocated to the local authorities (the city halls) for the enforcement of the law. Therefore, it is not surprising that the enforcement of the law was only partially successful with about half of P&B owners reporting not enforcing the law at all.

## Recommendation

Interventions including social marketing campaigns could encourage clients to request no-smoking enforcement in bars; this could bring owners to be more inclined to enforce the ban and more concerned that failure to adhere would cost them clients. This might be constructed in the context of ads that make clear that the majority of adults do not smoke and do not like eating or drinking in a smoke-filled bar, and that the majority should have the right to be protected from harm caused by a minority.

These policies are now in need of more formal and quantitative evaluation to extend the present qualitative study. As this is a qualitative study, the generalizability of the results is not clear, and the prevalence of the factors identified has not yet been quantified, even though a large number of towns and P&B were sampled. Further studies should try to understand why some P&B owners more than others implement the ban, using quantitative methodology, and why authorities have not enforced the law more vigorously. There is also a need to follow trends in smoking in P&B and quantitatively identify factors that can predict these changes. Deeper understanding of these factors may help implement more precise tobacco control policies. As smoking has generally decreased in Israel during the last few decades, it is possible to change social norms and behaviors in this society and prevent the morbidity and mortality due to smoking and SHS [34]. Lessons can be learned from this study on how to implement bans on smoking in Israel and in other countries based on the Israeli experience.

## Competing interests

The authors declared that they have no competing interest.

## Authors’ contributions

OB-E was involved in the conception and design of the study, wrote the manuscript, designed, and analyzed the data. CS was involved in the conception and design of the study, collected part of the data, and analyzed the data; in addition contributed to the drafting of the manuscript. VC collected part of the data, analyzed the data, and contributed to the drafting of the paper. AD-Z was involved in the conception and design of the study. MH was involved in the drafting of the manuscript. All authors read and approved the final manuscript.

## Authors’ information

**Orna Baron-Epel** is a professor at the School of Public Health, health promotion program, at the University of Haifa. Her research areas are health promotion, health behaviors, and social epidemiology.

**Carmit Satran** is a PhD candidate at the School of Public Health, Haifa University; this study is part of her doctoral dissertation.

**Vicki Cohen** is a health promoter and part of this study was her MPH thesis at the School of Public Health, Haifa University.

**Anat Drach-Zehavi** is an organizational psychologist and a senior lecturer at the Cheryl Spencer Department of Nursing, the Faculty of Welfare and Health Sciences, at the University of Haifa. Her area of research is leadership and teamwork in healthcare and evidence-based practice.

**Melbourne F. Hovell** is the Distinguished Professor and Director of the Center for Behavioral Epidemiology and Community Health, Graduate School of Public Health, San Diego State University. His research focuses on studies of the etiology of health-related behavior following the Behavioral Ecological Model. This includes studies of classic risk practices, such as smoking, diet, and physical activity, as well as studies of clinicians' service delivery.

## Funding

This study was funded by the Israel Cancer Association through the Environmental and Epidemiology Foundation of the late Israel Jacob and Lila Alther, and the Pfizer Public Health Policy Forum, University of Haifa, Israel.
